# Tunable uptake/release mechanism of protein microgel particles in biomimicking environment

**DOI:** 10.1038/s41598-017-06512-5

**Published:** 2017-07-20

**Authors:** A. Pepe, P. Podesva, G. Simone

**Affiliations:** 1 Northwestern Polytechnical University, 127 West Youyi Road, Xi’an Shaanxi, 710072 P.R. China; 2 University of Naples, Federico II, 80 Piazzale Tecchio, 80125 Naples, Italy

## Abstract

Microgels are intra-molecular crosslinked macromolecules that can be used as vehicles to deliver and release drugs at the point-of-need in the patient’s body. Here, gelatin microgels were formed from microfluidics droplets, stabilised by aldehydes and frozen into a spheroidal shape. Microgel morphology and response to external stimuli were characterised. It was found that the behaviour of the spheroidal microgels was sensitive to both pH and ionic strength and that the distribution of charges into the microgels affected the behaviour of swelling and uptake. The uptake of molecules such as Rhodamine B and Methylene Blue were investigated as a model for drug uptake/release mechanisms. Under physiological conditions, the uptake of Rhodamine was rapid and a uniform distribution of the fluorescent molecules was recorded inside the microgels. However, the mechanism of release became slower at lower pH, which mimics the stomach environment. Under physiological conditions, Methylene Blue release occurred faster than for Rhodamine. Anionic and neutral molecules were also tested. In conclusion, the dependence of uptake and release of model drugs on basic/acid conditions shows that microgels could be used for targeted drug delivery. Different shaped microgels, such as spheres, spheroids, and rods, could be useful in tissue engineering or during vascularisation.

## Introduction

Microgels, which constitute a new form of polymer, are structures with intra-molecular crosslinked macromolecules^1–3^. Over the years, microgels have gained a reputation as an intriguing class of polymeric materials; that is, they exhibit exceptional properties in regard to loading and releasing applications^4–7^. These properties derive from the unique combination of their colloidal nature (e.g. colloidal stability, high surface area, facile synthesis and control over particle size) with the inherent features of macroscopic hydrogels, i.e. their internal network structure (e.g. structural integrity in combination with fluid-like transport characteristics) and characterised by parameters such as mesh size, polymer volume fraction or interaction with embedded functional compounds. To date, the ability to control these factors by the application of external triggers represents the underlying concept of stimuli-responsive microgels^8, 9^.

Methods of microgel preparation are widely documented and range from solution bulk protocols^10^, intended as the formation and stabilisation in an emulsion, to the most recent based on microfluidics^11^, which enables a deep control on the dimensions and shape of the microgels as well as on their composition. In fact, special attention also has been given to the shape of microgels. The possibility to shape microgels with high control enables a wide variety of applications related to self-assembly. Assembling heterogeneous and homogeneous architectures from crosslinked microgel building blocks is in great demand in various applications, particularly in tissue engineering^12^ for mimicking biological functions and in the production of novel materials with engineered properties^13^.

Another intriguing aspect of these highly controllable processes refers to the fabrication of spherical and spheroidal microgels with anisotropic properties^14^. Indeed, anisotropic, injectable microgels could mimic local extracellular matrix architectures that cells encounter in complex tissues and could be extremely interesting for tissue engineering as well as for drug delivery.

Anisotropic hybrid microgels have been prepared using anisotropic particles as seeds for the later synthesis of responsive shells. Janus dumbbells^15^ or hematite and magnetite spindles^16, 17^ are some of the templates reported in the literature. Indeed, the design of non-spherical colloids makes them interesting candidates for self-assembly processes. Ideally, the key requirement towards complex assembled structures with desirable properties is the accurate engineering of colloidal particles. To qualify as suitable building blocks with anisotropy in shape and interactions, they need to be highly monodisperse in size and highly uniform in anisotropy. Finalised to biological applications, the preparation of the microgels starting from native biological raw materials (proteins, carbohydrates) resembles a crucial step for the advances in this field^18^. Indeed, thoroughly analysing the state of the art, many applications based on artificial materials or native mixed with non-native can be found^19, 20^.

Starting from that, we intend to fabricate microgels from native proteins, such as gelatin, with controlled shape and composition and characterise them when stimulated by sudden variations of the environmental conditions to mimic *in vivo* mechanisms. The variation of pH will simulate the mechanisms inside the intestinal tract or during digestion. To date, this will represent an optimal model to be pursued to design new suitable materials compatible with living organisms and useful for drug delivery applications.

## Experimental

### Materials

Gelatin (Dynamic viscosity: 1.5–6.0 mPa, bloom 300), Rhodamine B (Mw ∼479.02 g/mol), and glutaraldehyde (50%) were purchased from Sigma-Aldrich. Gelatin was used without further purification and prepared as a 15% wt solution at 50 °C. A three–stage Millipore Milli-Q plus 185 purification system with a resistivity higher than 18.2 MΩ was used.

### Fabrication of the microdroplet generator and microgels

The fabrication of the microgels in the microfluidic environment was reported elsewhere^21–23^. Briefly, the microfluidic chip had three inlets (ESI Fig. 1a) and the main channel had a T-junction for the generation of gelatin droplets. After 20 mm from the junction, the main channel broadened and connected to a fluidic element named *shuffling* ^21–23^, which, in turn, was connected to another inlet by a side channel. The width of the shuffling element was 200 μm; at the outlet of the shuffling element, the channel width was 100 μm. At the shuffling element, the droplets made by gelatin and the stream of crosslinker (1% in aqueous solution) mixed and the microgels spheroidal particles were formed. The gelatin was pre-loaded inside a reservoir.

For the experiments, the flow rate was controlled by high sensitivity pumps (Nemesys apparatus) and for the fluidic setup, glass syringes (1 mL and 2.5 mL) and PTFE tubing (0.8 mm ID) were used. The microfluidic device was housed vertically on an inverted microscope and the microgels were collected inside a Petri dish (ESI Fig. 1b). The flow stream was observed by the external camera and the inverted microscope worked for observing the microgels at the exit of the channel. Finally, polyimide mats connected to a power supply were used to heat the system up to 40 ± 5 °C.

Several flow rates were investigated before settling the operational conditions. After the trial investigation, the experiments were carried on at 0.8 μL/min: 1.5 μL/min: 1 μL/min for the system *gel: oil: glutaraldehyde*. To visualise the streams inside the channel, blue and red natural colours were used (ESI Fig. 1c).

The microgels were collected at the exit of the main channel (ESI Fig. 1c) and stored in acetone, which was also used for removing the residual oil coating the surface of the microgels.

### Characterisation of the microgels

#### SEM sample preparation

Scanning electron microscopy (SEM) was performed with a Zeiss Sigma apparatus (Zeiss, Italy) and TESCAN Vega3 (TESCAN, China). For both investigations, the samples were sputtered with gold for 2 min. To analyse the structure, the microgel particles were washed with acetone at least five times and then dried. The microgel section was obtained by cutting the particle with a surgical blade. The investigations were done using an Extra-High Tension (EHT) = 16 kV.

#### Ionic strength and pH on the size and permeability

To explore microgel behaviour in response to different external stimuli, the microgels first were extracted by acetone and incubated in deionised water. After stabilisation in water, the microgel samples were added to NaCl solutions ranging in concentration from 0.25 M to 1 M. An analogous protocol was followed for studying response to pH change. In particular, HCl and buffered solutions were used to set the pH range of the microgels.

For both ionic- and pH-conditioned solutions, the analysis was done by controlling and recording the weight of the samples as well as the characteristic dimensions. The latter set of data was obtained by observing the samples at set timeframes by inverted microscope (Olympus, Italy) and analysing the images with ImageJ software.

#### Zeta potential

The zeta potential of the microgels was measured by varying the pH in the Zetasizer (Malvern, China available at The Key Laboratory of Space Applied Physics and Chemistry, Northwestern Polytechnical University, Xi’an). About 3 mL of the suspension (1 mg/mL) was added to a cuvette and adjusted to pH values in the range from 2–10 using HCl and NaOH. The suspension was equilibrated for 4 min at 25 °C. The measurement was performed with three runs, with each run consisting of 10 single measurements.

#### Uptake and release

The uptake of Rhodamine B was achieved by incubating the microgels for 10 h in a solution with 0.1 mg/mL. The gelatin microgels were loaded with Rhodamine B in buffer solution pH 7.4 at 37 °C, while the release was carried at pH 7.4 and pH 6.7. In both cases, they were rinsed in water three times to remove excess fluorescent tag. Spectrometer combined with an optical investigation (Olympus inverted microscope) was used to estimate the release. After each washing step, the supernatant was withdrawn from the samples; in turn, fresh buffer was added for the new interval of incubation. The absorbance of Rhodamine B at λ = 540 nm was recorded to determine the calibration curve of the absorbance spectrum. Following, the released amount was calculated for each measurement with concentrations extrapolated by the Rhodamine calibration curve.

For estimating the mechanism of release of Methylene Blue (MB) the microgels were incubated with MB solution (12 μM, pH 7.4) for 24 h. To measure the mechanism of release, the microgels were washed and then incubated in buffer at pH 7.4 for 30 h. After each washing step, the supernatant was withdrawn from the samples; in turn, the same volume of fresh buffer was added for the new interval of incubation. During the time of the experiment, the absorbance of MB in buffer was measured using a spectrometer (Ocean Optics HR4000, China) at a wavelength of 580 nm. The amount of MB was determined from the calibration curves from the readout of the solution at several dilutions. The absorbance of the samples at different times of incubation provided the amount of the MB released at the sampling time.

Uptake/release mechanisms of Fluorescein and Indocyanine Green were studied as well. For estimating the mechanism of release of Fluorescein (332.1 g/mol) and Indocyanine Green (774 g/mol), the microgels were incubated with the dye solutions (10^−4^ g/mL μM) for 24 h. To measure the mechanism of release, the microgels were washed and then incubated in buffer at pH 7.4 and 6.7 for 30 h. After each washing step, the supernatant was withdrawn from the samples; in turn, the same volume of fresh buffer was added for the new interval of incubation. During the time of the experiment, the absorbance of Fluorescein and Indocyanine Green in buffer was measured at a wavelength of 490 nm and 680 nm, respectively. The amount of dyes was determined from the calibration curves from the readout of the solution at several dilutions. The absorbance of the samples at different times of incubation provided the amount of the dyes released at the sampling time. It worth observing that an absorption and fluorescence properties of Fluorescein depends on the pH of the solution^24^; to date, before the measurements, the pH was adjusted with NH_3_. The calibration curve was built under the same conditions.

## Results and Discussion

### Microgel morphology

The microgel fabrication design had the aim to collect spheroidal particles. In fact, anisotropic and non-spherical microgels could influence the response of biological systems (i.e. cells)^25^.

**Figure 1:**
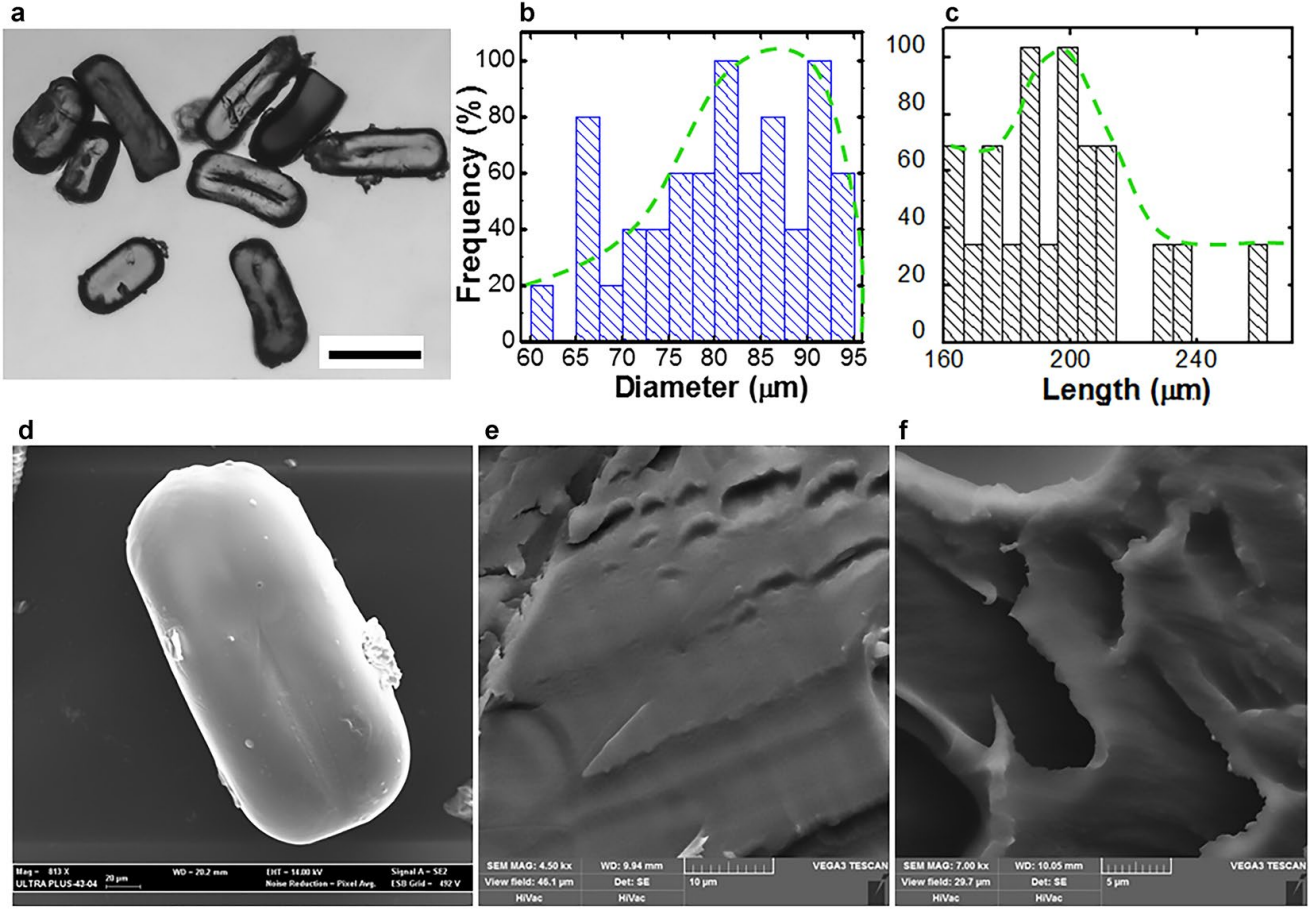
Microgel characterisation. (**a**) Sample of spheroidal microgels washed with acetone and dried. Bar: 100 μm. (**b**) Diameter distribution of the microgels and cumulative distribution (dotted line). (**c**) Length distribution of the microgels and cumulative distribution (dotted line). (**d**) SEM image of the single spheroid microgel. (**e**) Microgel internal structure. (**f**) Microgel internal microstructure with cavities.

After fabrication, the stabilised microgels were collected at the exit of the microfluidic channel, washed and finally stored at room temperature in acetone for several days (Fig. 1a). The size distribution, as derived by the analysis of the microscope images, is shown in Fig. 1b and c according to the microgel diameter and length. The diameter distribution had a maximum at μ = 80 ± 2.5 μm and an average size of 60 ± 8.9 μm; besides, the length distribution had a maximum at μ = 200 ± 10.1 μm and an average size 180 ± 9.3 μm.

Following, the microstructure of the microgels was analysed. Despite several washings with acetone, scanning electron micrographs of the microgels did not show surface defects or collapses (Fig. 1d). The internal structure of the microgels was detected to be nonporous and homogenous (Fig. 1e); while a tiny percentage of microgels (<10%) displayed a significant porosity (Fig. 1f). Nonetheless, the observed porosity was not well structured or interconnected. In fact, some instabilities recorded during the fabrication process could have caused the porosity of the microgels, such as the air bubbles trapped inside the gelatin.

### Ionic strength

**Figure 2:**
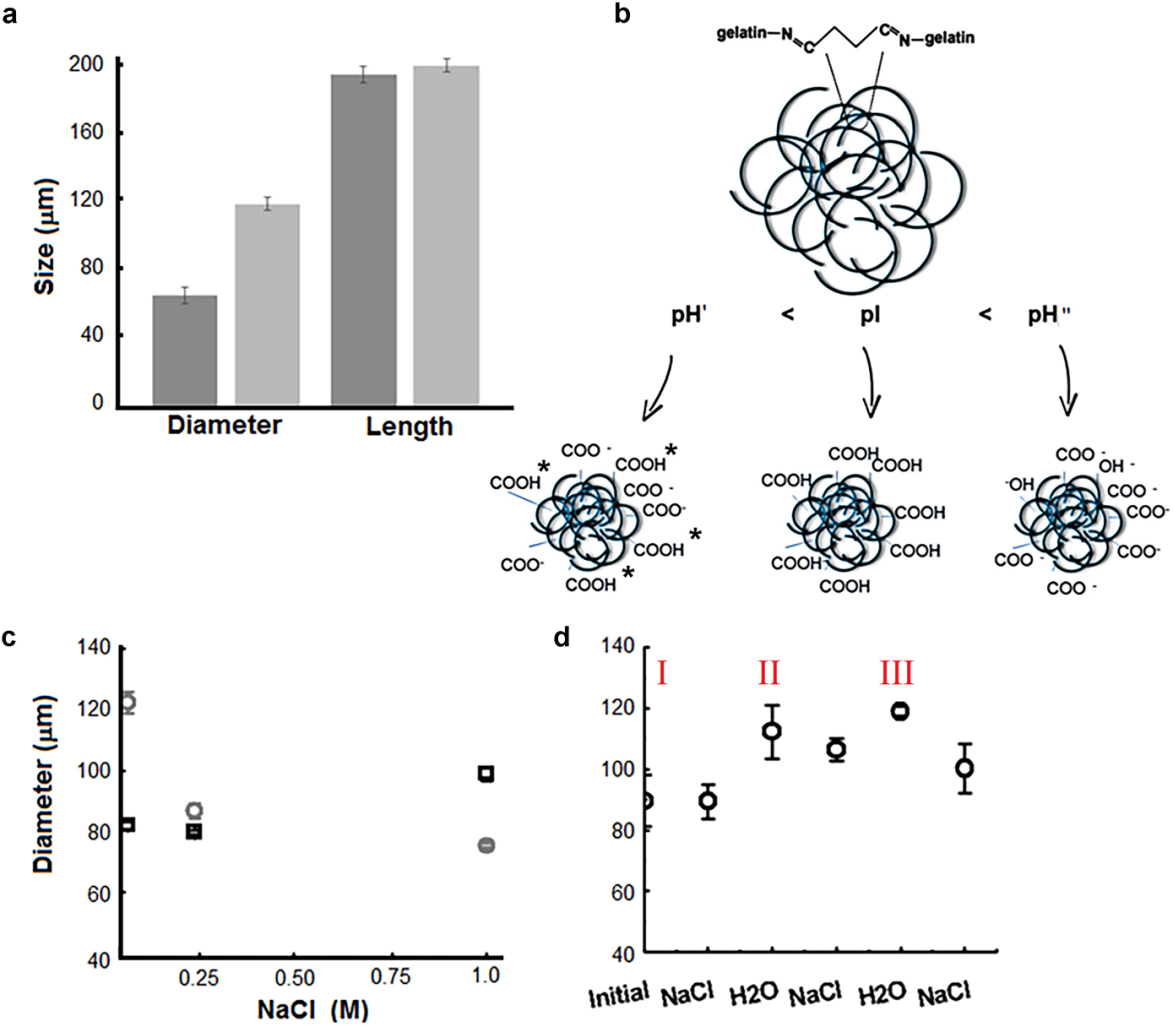
(**a**) Size of the microgels (diameter and length) after three days in water. Darker columns represent the initial state, clearer the final state. (**b**) Cartoon displaying the gelatin charge distribution at different pH conditions and at pH = pI. *Symbol highlights the protonated anions at pI. (**c**) Diameter variation according to the concentration of NaCl in the suspending solution. The initial conditions are recorded after 3 days swelling in water (panel a), [NaCl] = 0. Symbols of scatter plot: ○ at pH 7.4, □ at pH = pI. (**d**) Cyclic change of the ion concentration of the incubation buffer. The label in Romanic evidences the cycle number. The concentration of NaCl is 0.5 M.

Several overlapping chemical – physics mechanisms can influence the microgel behaviour and properties. An essential behaviour of microgels is swelling/shrinking in aqueous/dry environments in response to external stimuli^26^. Here, the microgels were suspended in water and the swelling was measured by recording the dimensional variations (Fig. 2a). After three days, the diameter was measured to be about 110.4 ± 8.4 μm and the moderate mechanism of water uptake was explained as the consequence of the scarce porosity of the microgels, according to the results discussed above. However, other intrinsic properties can motivate this behaviour as well. The mechanisms of capturing and trapping water and aqueous solutions was studied as an effect of the zwitterionic behaviour of the gelatin. The gelatin is a protein and its isoionic point has been recorded at pH 5. To date, the role of pH in affecting the absorption of aqueous solutions is a crucial argument to be understood.

Two microgel samples were suspended in aqueous solutions at pH 7.4 (pH > pI) and pH 5 (pH = pI). At the end of the incubation time, the two samples displayed different diameters: 120 μm for microgels stabilised at pH 7.4 and 85 μm for microgels stabilised at pH = pI. Then, the microgels were dried for seven days at room temperature and their weight was recorded. The samples were re-suspended in aqueous solutions and the pH adjusted to 7.4 and 5, respectively. After three days, from dry to swelling equilibrium, the weight of the microgels suspended at pH 7.4 increased from 140 μg to 360 μg, whereas the weight of the microgels suspended at pH 5 increased from 140 μg to 230 μg. To date, the swelling capability of the microgels resulted increased at pH higher than pI.

The charge of the crosslinked microgels was investigated by measuring the zeta potential (ζ) according to the pH. It was recorded that the zeta potential of the microgels swollen in a buffer at pH 7.4 resulted in ζ = −13.2 ± 0.5 mV. The negative value of the zeta potential was explained as the consequence of crosslinking reactions. The glutaraldehyde links with the amine groups of the gelatin by reaction of condensation, while the carboxylic acid groups remain in the native state and generate negative charges at the pH 7.4. At pH 8.5, the zeta potential was measured to be ζ = −30.1 ± 0.6 mV. Upon increasing the pH, the amine groups not involved with the condensation reaction deprotonate and, indirectly they become additional sources of negative charge.

In conclusion, at pH ≫  pI, the carboxylate and amine groups, according to pH strength, were ionised and generated negative charges distributed along the chains. The negative charges enhanced electrostatic anion–anion repulsions and caused an improved swelling capacity^27^.

At the pI, the zeta potential is about null and the microgels displayed a modest swelling^28, 29^. In fact, at this pH, the carboxylate anions and the amine groups not linked with the glutaraldehyde in the reaction of crosslinking are protonated, so that the microgels charge resulted almost null.

Under acid conditions, at pH 4 (pH < pI), the zeta potential was measured to be ζ = −6.5 ± 0.3 mV as consequence of the partial protonation of the carboxylate anions and reduction of the anion–anion repulsion, which led to a remarkable decrease in swelling capacity. Figure 2b displays a cartoon that highlights the charges available inside the microgels at different pH values.

Following, the microgels at pH 7.4 and pI were suspended in NaCl solutions with concentrations ranging from 0 to 1 M (Fig. 2c). The microgels stabilised at pH = pI augmented their size according to the ionic strength. Conversely, a divergent trend was recorded at pH > pI, where the microgels shrunk by increasing the ionic strength of the suspending solution. At pH 7.4, the shrinking of the microgels has been argued as a consequence of an anti-polyelectrolyte swelling behaviour. In suspension in low ionic strength media, the charges of the microgels presented a high level of attraction and led to the collapse of the network. The hypothesis could be that, with the addition of the ion salt, the attractive interactions inside the microgel were screened and the ionic bonds destroyed and replaced by the electrostatic interaction with the cations^29^. However, the salt ions hindered the molecules of water leading the shrinking of the microgels with the increase of the salt concentration in solution.

The isoionic microgel was able to capture either positive or negative ions through electrostatic interactions, with the addition of ion salt in solution. The charges distributed along the chains and inside the microgels increased; as a result, the free negative charges available on the chains of the protein were able to retain water while the microgels swelled.

As a consequence of the last phenomenon, the microgels underwent an anisotropic swelling along the two main directions (length and diameter)^30^. In fact, this anomalous behaviour of the microgels could be the consequence of network constrictions formed inside the protein structure, which reduced the capability of the microgels to grasp the water molecules and constrained the movement of the chains^31^.

The cyclic and repeatable response to external stimuli is an important aspect for the microgels in both drug loading and release mechanisms. To test this property, the same microgels were incubated in NaCl solution (0.5 M) and aqueous solution at pH 6.7, by turns. Figure 2d displays three cycles (labelled I, II, and III). Starting from the initial point, after the stabilisation of the microgels in saline solution, the diameter of the microgels remained almost unaltered. From the salt solution to water the diameter of the microgels rise from 80 μm to 110 μm. The incubation in water swollen the microgels, but at the same time reset the organisation of the polymeric chains that did not allow the particles to reach native size after each cycle.

In fact, during cycle II, it was observed that changing the environment from water to salt did not bring the microgels to the native size, whereas the recurring behaviour of the microgel properties was observed starting from the cycle II onward.

### Uptake and release of drug-like molecules

An important application of microgels is in the field of nanomedicine, such as the uptake/release of molecules for effective therapies. Here, the spheroidal microgels were investigated for the uptake/release of Rhodamine B and MB, common payload-model molecules for drug delivery applications. These molecules are interesting models to investigate as they recently have been shown to selectively induce apoptosis in cancer cells^30, 31^. Furthermore, Rhodamine and MB have a positive charge and low molecular weight; therefore, they are perfect models for drugs^32^.

The microgels were incubated with Rhodamine B according to the conditions reported above and the analysis was carried out analysing both the microgels and supernatant. The release was carried at pH 7.4.

**Figure 3:**
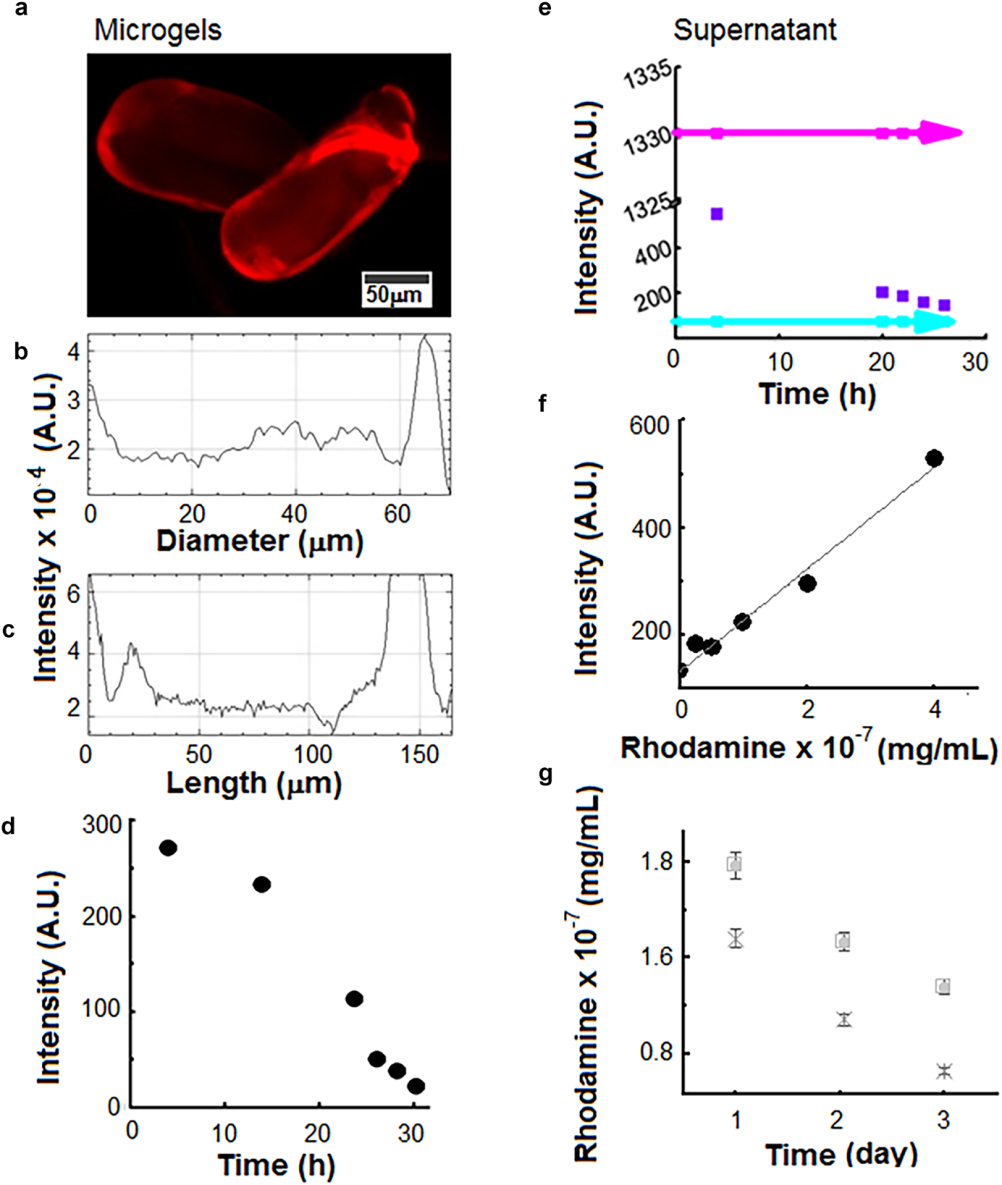
(**a**) Microgels in Rhodamine B. Bar: 50 μm. (**b**) Intensity along the diameter of the microgel. The two peaks at the sides of the profile are prompted to the higher intensity recorded at the border. (**c**) Intensity along the length of the microgel. As above, border effects are present. (**d**) Spectrometric characterisation of the microgels during the 30 h of payload release. (**e**) Spectrometric characterisation of the supernatant withdrawn during the washing time. The arrows describe the pure Rhodamine (top) and the buffer (bottom) profiles used as blanks. (**f**) Calibration curve correlating the Rhodamine B amount and the emitted fluorescence. (**g**) Quantification of the payload release of Rhodamine B. The experiments were run three times under identical environmental conditions. Error bars show the standard deviation. Symbols of scatter plot: □: pH 7.4, ×pH 6.7.

A micrograph of the microgels is displayed in Fig. 3a while Fig. 3b and c highlight the spatially resolved intensity profiles of the red fluorescence in the single particle. After the time of the incubation, the microgels emitted a uniform signal along both width and length, while the peaks of fluorescence were observed at the edges. During the time when the release was studied, the microgels were tracked and the spectrometer signal over time was recorded. Figure 3d shows that the intensity of fluorescence versus the time dropped with a linear trend from 250 A.U. down to 10 A.U.

During the same timeframe, the supernatant withdrawn from the tubes was examined. The curve of the emission of the supernatant is plotted versus the time and compared with the spectra of the fresh buffer and Rhodamine (0.1 mg/mL, 200 μM) (Fig. 3e). In less than 30 h, the fluorescence signal dropped from 1325 A.U. to 100 A.U. The samples were analysed at the microscope as well. First, a calibration curve was determined to correlate the payload concentration to the emitted signal (Fig. 3f). Then, the supernatant samples were analysed (Fig. 3g). It was observed that after 30 h, the gelatin microgels had some residual content of Rhodamine (6 × 10^−8^ mg/mL), which lead to conclude that in almost one day, the payload was delivered inside the surrounding environment. The release of Rhodamine was also studied at pH 6.7 to display that raising the pH of the suspending solution, the permeability of the microgels increased due to higher charge repulsion, thereby allowing the faster release of Rhodamine B from the microgels.

To mimic a gastric acid environment, the release of Rhodamine was analysed at pH 3.5. Table 1 reports the values of the released Rhodamine after 24 h and 40 h according to the pH. Under acid conditions, the mechanism of release resulted slower, due probably to a mechanism of negligible charge repulsion, which therefore keeps the payload inside. Table 1 summarizes the percentage of released Rhodamine under different environmental conditions according to the time.

**Table 1:** Percentage of release Rhodamine^§^, %.

Release time, hours	pH 3.5	pH 5	pH 7.4
*5*	70	79	72
*24*	92	98	96
*48*	94	99	98

^§^Calculated on 140 μg of upload Rhodamine B in 1 mg of microgels.

In conclusion, the mechanism of release is also affected by the charge distribution and, as consequence, the drug release can be controlled by variations in pH or the medium’s ionic strength. The pH-dependent behaviour makes the gelatin an interesting biomolecules for drug delivery. In fact, the gelatin microgels are excellent carriers for drugs, as they can protect the drugs from undesired interactions with gastric fluids and release them when they reach the target cells.

**Figure 4:**
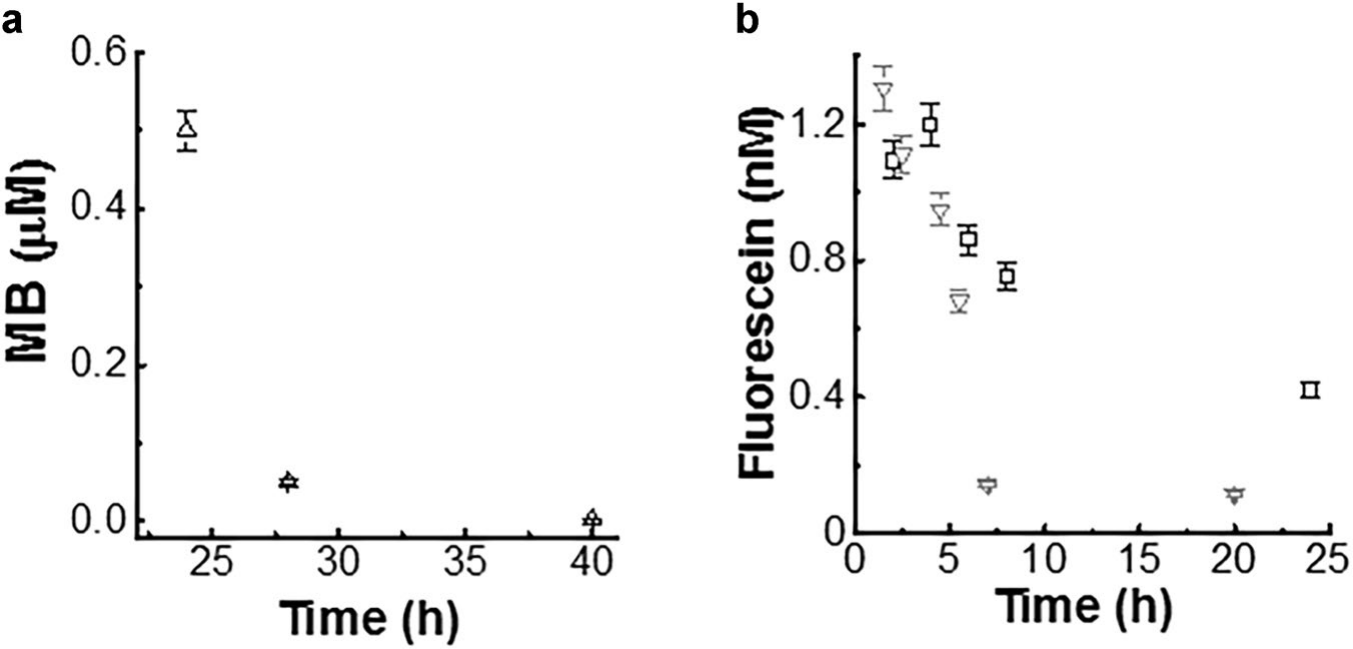
(**a**) Quantification of MB-payload release at pH = 7.4. (**b**) Quantification of Fluorescein-payload release. Error bars show the standard deviation. Symbols of scatter plot: □: pH 7.4, ▽: pH 6.7.

The mechanism of release of drugs was also mimicked with MB according to the protocol used with Rhodamine. In particular, drug release efficiency was investigated at pH 7.4. The concentration of the payload released over time is displayed in Fig. 4a (ESI Fig. 2).

The MB released within the first 24 h of incubation brings the concentration of released Rhodamine to 0.50 μM; however, after 28 h, the concentration of MB released by the microgels reduced to 0.05 μM and reached a value of 2 × 10^−4^ μM after about 40 hours. Comparing the value of Rhodamine and MB (each used with different initial concentrations) at physiological pH, the mechanism of release of MB was faster than for Rhodamine. In fact, the initially loaded concentrations of the molecules differed by one order of magnitude (200 μM versus 12 μM) while the payload concentrations in solution were 0.1 μM Rhodamine versus 2 × 10^−4^ μM MB. However, Rhodamine molecules are bigger than MB ones, to date, the faster release of the latter can be a consequence of the diffusion mechanism inside the gelatin network.

To understand the behaviour of the gelatin microgels in presence of negative and neutral dye molecules, fluorescein and indocyanine green release was examined at pH 7.4 and at pH = 6.7. The quantification of fluorescein payload released over time is displayed in Fig. 4b. The concentration of the released neutral payload resulted to be at least two-order-of-magnitudes lower than the MB one.

The Fluorescein molecules permeated the gelatin by diffusion; however, the mechanism of release is quite fast and it ended less than 7 h, when more than 98% of the payload was released^31^. The latter result confirmed that the Fluorescein permeating the network was not anchored to the chains, due to the absence of electrostatic interactions and of the ionic effects.

In contrast, it was impossible to detect a significant amount of uploaded and released Indocyanine Green (ESI Fig. 3), the negative dye. The previous results lead to the conclusion that while the Fluorescein can diffuse inside the network of the gel, the molecules of Indocyanine Green are repelled by the electrostatic forces, having the same charge of the crosslinked gelatin. It worth mentioning that the Indocyanine Green molecules is double than Fluorescein ones, and, consequently, the mechanism of diffusion was also negligible and supporting the validity of the observed results.

In conclusion of this section, biodegradability and biocompatibility are essential properties for materials used in drug delivery. Microgels can be digested in a few hours in the presence of trypsin^21^.

## Conclusions

The present work details an in-depth investigation and characterisation of natural microgels made from native, chemically stabilised proteins after preparation in a microfluidics environment.

In particular, an accurate analysis of the results showed that microgels have uniform size distribution while displaying anisotropic swelling. The microgels display different responses to environmental stimuli, e.g. swelling or shrinking under different conditions of pH and ionic strength. These properties were explained in light of the chain and charges available in the gelatin network.

Finally, using Rhodamine and MB as payload models, the uptake/release mechanisms were recorded in conditions that mimicked the release of a drug in humans. The combination of unique shape and response behaviour documented here suggest that these microgels could be used for tissue engineering. Furthermore, by controlling the microgels’ shape (i.e. sphere, spheroid, or rod) one can control the direction of cell growth as well as drug delivery.

Finally, it worth concluding that the simplicity of microgel fabrication, which is a one-step process, makes the protocol easily scalable.

## Electronic supplementary material

Supplementary

## References

[1] Oh JK, Drumright R, Siegwart DJ, Matyjaszewsk K (2008). The development of microgels/nanogels for drug delivery applications. Progress in Polymer Science.

[2] Hoffman AS (1987). Applications of thermally reversible polymers and hydrogels in therapeutics and diagnostics. J Control Release.

[3] Peppas NA, Hilt JZ, Khademhosseini R, Langer R (2008). Hydrogels in biology and medicine: from molecular principles to applications. Adv. Mater..

[4] Haraguchi K, Song L (2007). Microstructures Formed in Co-Cross-Linked Networks and Their Relationships to the Optical and Mechanical Properties of PNIPA/Clay Nanocomposite Gels. Macromolecules.

[5] Madsen J, Armes SP, Lewis AL (2006). Preparation and Aqueous Solution Properties of New Thermoresponsive Biocompatible ABA Triblock Copolymer. Macromolecules.

[6] Li S, El Ghzaoui A, Dewinck E (2005). Rheology and drug release properties of bioresorbable hydrogels prepared from polylactide/poly(ethylene glycol) block copolymers. Macromol Symp..

[7] Park SY, Lee Y, Bae KH, Ahn CH, Park TG (2007). Macromol Rapid Commun..

[8] Ajayaghosh A, Varghese R, Praveen VK, Mahesh S (2006). Evolution of Nano‐to Microsized Spherical Assemblies of a Short Oligo (p‐phenyleneethynylene) into Superstructured Organogels. Angew Chem Int Ed, 2006.

[9] Deng W, Yamguchi H, Takashima Y (2007). A chemical-responsive supramolecular hydrogel from modified cyclodextrins. Angew Chem Int Ed..

[10] Wang LY, Gu YH, Zhou QZ, Ma GH (2006). Preparation and characterization of uniform-sized chitosan microspheres containing insulin by membrane emulsification and a two-step solidification process. Colloids Surf B: Biointerfaces.

[11] Zhang H, Tumarkin E, Sullan RMA, Walker GC, Kumacheva E (2007). Exploring Microfluidic Routes to Microgels of Biological Polymers. Macromol Rapid Commun.

[12] Du Y, Lo E, Ali S, Khademhosseini A (2008). Directed assembly of cell-laden microgels for fabrication of 3D tissue constructs. Proc. Natl. Acad. Sci. USA..

[13] Eng G (2013). Assembly of complex cell microenvironments using geometrically docked hydrogel shapes. Proc. Natl. Acad. Sci. USA..

[14] Kim JW, Larsen RJ, Weitz DA (2006). Synthesis of nonspherical colloidal particles with anisotropic properties. J. Am. Chem. Soc..

[15] Zhang ZK, Krishna N, Lettinga MP, Vermant J, Grelet E (2009). Reversible gelation of rod-like viruses grafted with thermoresponsive polymers. Langmuir.

[16] Hoffmann M (2010). Thermoresponsive colloidal molecules. Soft Matter.

[17] Dagallier C, Dietsch H, Schurtenberger P, Scheffold F (2010). Thermoresponsive Hybrid Microgel Particles with Intrinsic Optical and Magnetic Anisotropy. Soft Matter.

[18] Tang MD, Golden AP, Tien J (2003). Molding of three-dimensional microstructures of gels. J. Am. Chem. Soc..

[19] Du JZ, Sun TM, Weng SQ, Chen XS, Wang J (2007). Synthesis and characterization of photo-cross-linked hydrogels based on biodegradable polyphosphoesters and poly (ethylene glycol) copolymers. Biomacromolecules.

[20] Li Q, Wang J, Shahani JS, Sun DDN (2006). Biodegradable and photocrosslinkable polyphosphoester hydrogel. Biomaterials.

[21] Simone G (2015). Demonstrating microdroplet coalescence for tailored and biodegradable microgel fabrication. RSC Advances.

[22] Simone G (2016). An alternative approach to the phase change of proteins in an aqueous mixture with ethanol. Chemical Engineering Research and Design.

[23] Simone G, Netti PA (2013). Non-spherical gelatin particle in two phases microfluidic system. Microelectronic Engineering.

[24] Sjöback R, Nygren J, Kubista M (1995). Absorption and fluorescence properties of fluorescein. Spectrochimica Acta Part A: Molecular and Biomolecular Spectroscopy.

[25] Harley BAC, Kim HD, Zaman MH, Yannas IV (2008). Microarchitecture of three-dimensional scaffolds influences cell migration behavior via junction Interactions. Biophysical Journal.

[26] Flory, P. J. Principles of polymer chemistry. Ithaca, NY: Cornell University Press (1953).

[27] Wen X (2015). Preparation of CMC/HEC Crosslinked Hydrogels for Drug Delivery. BioResources.

[28] Taleb MFA, El-Mohdy HLA, El-Rehim HAA (2009). Radiation preparation of PVA/CMC copolymers and their application in removal of dyes. J. Hazard. Mater..

[29] Duconseille A, Astruc T, Quintana N, Meersman F, Lhoutellier S (2015). Gelatin structure and composition linked to hard capsule dissolution: A review. Food Hydrocolloids.

[30] Oh JK, Lee DI, Park JM (2009). Biopolymer-based microgels/nanogels for drug delivery applications. Progress in Polymer Science.

[31] Flory PJ (1941). Molecular size distribution in three dimensional polymers. I. gelation. J. Amer. Chem. Soc..

[32] Glotzer SC, Solomon MJ (2007). Anisotropy of building blocks and their assembly into complex structures. Nature Materials.

