# Supplementary material for: Tunable uptake/release mechanism of protein microgel particles in biomimicking environment
Source: Sci Rep. 2017 Jul 20;7:6014. doi: 10.1038/s41598-017-06512-5 (PMC5519546; doi:10.1038/s41598-017-06512-5)
Supplement: Supplementary file 1 — Supplementary [file 41598_2017_6512_MOESM1_ESM.pdf]

Tunable uptake/release mechanism of protein microgel particles in biomimicking environment

A. Pepe,[a] P. Podesva,[b] G. Simone,[b,a]

[a] University of Naples, Federico II, 80 Piazzale Tecchio, 80125 Naples, Italy

[b] Nothwestern Polytechnical University, 127 West Youyi Road. Xi'an Shaanxi, 710072, P.R.China

Email: giuseppina.simone@nwpu.edu.cn; Tel. +86 150 2904 0025.

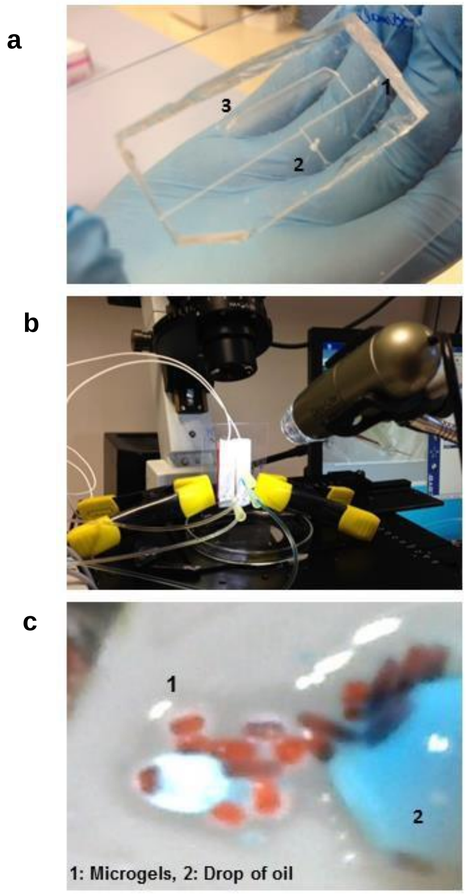

Fig. 1. a. The microfluidic device is use for the experiments. The numbers from 1 to 3 refer to the inlets to the microchannels. 1 oil, 2 stabilizer and 3 gelatin; **b.** The setup of the experiments includes the vertical positioning of the device controlled by an external camera, and a Petri dish where the microgels are collected monitored by the inverted microscope; resistive patches permit the control of the temperature of the device; **c.** the microgels were collected in dishes mixed with the carrier oil. The numbers embedded inside the figure highlight the microgels and the oil, while the colors are due to the tracers used for the experiments.

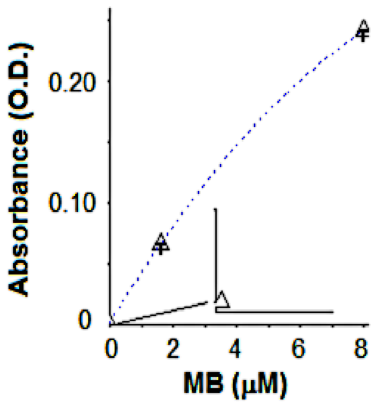

Fig. 2. Calibration curve correlating Methylene Blue concentration and the emitted signal detected by the spectrometer at 580 nm. The inset magnifies the absorbance at 2×10^{-4} μM.

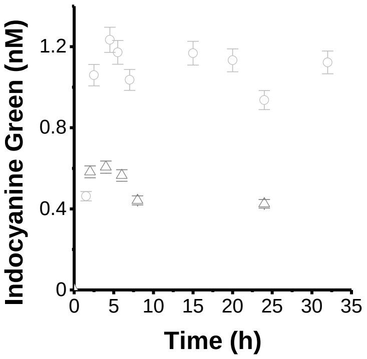

Fig. 3. Quantification of Indocyanine Green-payload release. Error bars show the standard deviation. Symbols of scatter plot: ○ : pH 7.4, Δ : pH 6.7.
